# Expression Analysis of Sound Vibration-Regulated Genes by Touch Treatment in *Arabidopsis*

**DOI:** 10.3389/fpls.2017.00100

**Published:** 2017-01-31

**Authors:** Ritesh Ghosh, Mayank A. Gururani, Lakshmi N. Ponpandian, Ratnesh C. Mishra, Soo-Chul Park, Mi-Jeong Jeong, Hanhong Bae

**Affiliations:** ^1^Department of Biotechnology, Yeungnam UniversityGyeongsan, South Korea; ^2^Department of Biology, College of Science, United Arab Emirates UniversityAl Ain, United Arab Emirates; ^3^National Institute of Agricultural Sciences, Rural Development AdministrationWanju, South Korea

**Keywords:** sound vibration, touch, mechano-stimulus, MS ion channel, electrolyte leakage, photosynthesis

## Abstract

Sound vibration (SV) is considered to be a mechanical stimulus which gives rise to various physiological and molecular changes in plants. Previously, we identified 17 SV-regulated genes (SRGs) which were up-regulated by SV treatments in *Arabidopsis*. Here, we analyzed the expression pattern of similar genes after an exposure of 500 Hertz at 80 decibels, for various time periods. Simultaneously, we confirmed the SV-mediated expression of these genes under lighted condition as many of them were reported to be dark-induced. For this, we designed an improved SV treatment chamber. Additionally, we checked the electrolyte leakage (EL), photosynthetic performance and expression of mechanosensitive (MS) ion channel genes after 5 days of SV treatment in the illuminated chamber. EL was higher, and the photosynthetic performance index was lower in the SV-treated plants compared to control. Seven out of the 13 MS ion channel genes were differentially expressed after the SV treatment. Simultaneously, we checked the touch-mediated expression pattern of 17 SRGs and 13 MS ion channel genes. The distinct expression pattern of 6 SRGs and 1 MS ion channel gene generate an idea that SV as a stimulus is different from touch. Developmental stage-specific expression profiling suggested that the majority of the SRGs were expressed spatiotemporally in different developmental stages of *Arabidopsis*, especially in imbibed seed, seedlings and leaves.

## Introduction

Sound vibration (SV) is considered to be a mechanical stimulus which can create the thigmomorphogenetic response in plants (Telewski, 2006). Available evidences suggest that the interaction between SV and plants is relevant both in ecological as well as environmental context. For instance, the phenomenon of ‘Buzz Pollination’ has been noted in number of plant species which indicates the ecological relevance of SV. Buzz- pollinated plants release pollen from anthers only at a particular frequency produced by bee’s buzz (De Luca and Vallejo-Marin, 2013). Similarly, plants’ responsiveness to environmental sound has been shown recently. Pretreatment with vibrations caused by chewing sound of caterpillar has been noted to elicit plant defense against herbivore (Appel and Cocroft, 2014). This advocates the relevance of natural SV in plants’ defense. In addition, several other plausible environmental significance of SV has been discussed by Mishra et al. (2016). Taken together, it is amply clear that like other physical factors SV is ecologically and/or environmentally significant to plants.

Experimentally, previous studies have shown the various effects of synthetic single frequency SV on plants (Hassanien et al., 2014). SV has the ability to alter antioxidant activities, calcium flux, sugar and ATP contents, hormonal modulation and plasmalemma architecture in plants (Mishra et al., 2016). SV-induced antioxidant (like- catalase, superoxide dismutase, and ascorbate peroxidase) activity was noted in chrysanthemum seedlings and hazel cells (Xiujuan et al., 2003; Safari et al., 2013). Increased ATP content in the SV-treated *Actinidia chinensis* callus suggests that SV can alter the energy metabolism (Xiaocheng et al., 2003). SV-induced changes in levels of phytohormones (like- auxin, cytokinin, and salicylic acid) were previously reported in *Arabidopsis* plant, *Chrysanthemum* callus, and protocorm-like bodies of *Dendrobium* (Bochu et al., 2004; Wei et al., 2012; Ghosh et al., 2016). Besides that, beneficial effects of SV were noted in terms of disease resistance, crop yield, callus regeneration and plant growth (Hassanien et al., 2014). A growing body of recent evidence suggests the existence of sophisticated molecular mechanisms for SV perception and signal transduction in plants. Despite this, however, there exists a huge gap in our understanding regarding the SV-mediated molecular alterations in the cellular milieu, which is a prerequisite to gain insight into SV-mediated plant development. Necessitated by this, we had previously investigated the global transcriptomic and proteomic changes in *Arabidopsis thaliana* upon treatment with SV of five different frequencies (250, 500, 1000, 2000, 3000 Hz) with constant amplitude to bridge this gap (Ghosh et al., 2016). Several genes were noted to be differentially expressed after SV treatment, and it was noted that 500 Hz for 1 h has maximum impact on cellular processes in *Arabidopsis*. Additionally, 17 genes were further confirmed by real-time PCR analysis, which are termed as *S*V-*r*egulated *g*enes (SRGs).

Another well-known mechanical stimulus which alters the physiology of plants at various levels is touch. Touch-mediated growth retardation, calcium spiking, reactive oxygen species generation, hormonal modulation and gene expression were previously reported (Braam, 2005; Chehab et al., 2011). It has been demonstrated that repetitive touch treatment can prime plants, which subsequently alter the plant defense against fungal pathogen and herbivore (Chehab et al., 2012). Previously, we hypothesized that SV and touch share some common mechanosensitive (MS) signaling events, as many of the touch-regulated genes were induced by SV (Ghosh et al., 2016). Therefore, it was concluded that touch-mediated expression profiling of SRGs is necessary to strengthen this idea. In the present study, we investigated the expression of SRGs after the touch treatment. Simultaneously, we checked the expression pattern of similar genes after exposure of 500 Hz for various time periods, and investigated their transcript levels in various developmental stages. Additionally, we have designed an improved SV treatment chamber, and cross-confirmed the SV-mediated expression of SRGs in lighted conditions. Simultaneously, this chamber was used to investigate the effect of long-term SV treatment on physiological responses like electrolyte leakage (EL) and photosynthesis.

It was hypothesized that plant MS ion channels have important roles in mechanical stress perception and signaling (Monshausen and Haswell, 2013; Mishra et al., 2016). In plants, broadly, three major groups of MS ion channels were reported: MS channel of small conductance-like (MSL), Mid1-complementing activity family (MCA) and Piezo (Monshausen and Haswell, 2013). MSLs in *Arabidopsis* are homologous to *Escherichia coli* MS channel of small conductance (MscS) protein which provides the rapid release of osmolytes from cells in response to the increased membrane tension. MCA and Piezo belong to the putative stretch-activated Ca^2+/-^permeable channels. Various electrophysiological, pharmacological and molecular-genetic analyses identified the importance of MSL9, MSL 10 and MCA1 for mechanosensitive activity in *Arabidopsis* (Nakagawa et al., 2007; Haswell et al., 2008). Additionally, the functions of MSLs in various biological processes, like- chloroplast shaping, cell death signaling, and pollen germination, were previously reported (Haswell and Meyerowitz, 2006; Veley et al., 2014; Hamilton et al., 2015). Here, we investigated the expression level of MS ion channels after 5 days of continuous SV treatment. Simultaneously, we checked the transcript levels of these MS channel genes after repetitive touch treatments for 5 days.

In summary, we investigated the expression of 17 SRGs and 13 MS-ion channel genes after SV and touch treatments. Simultaneously, we have checked the developmental stage-specific expression pattern of 17 SRGs. In addition, EL and photosynthetic performance were analyzed to check the SV-mediated physiological response after the long exposure.

## Materials and Methods

### Plant Materials and Growth Condition

*Arabidopsis thaliana* (Col-0 ecotype) seeds were placed in pots containing artificial soil (Punong, Korea) and stratified for 2 days in the dark at 4°C for homogenous germination. Subsequently, pots were transferred to the growth room and seedlings were allowed to grow under continuous light (∼150 ± 10 μmol m^-2^ s^-1^) at 22 ± 1°C. The growing seedlings received supplements with nutrients (Bio-nex, Korea) mixed in water at every 3-day interval.

### SV Treatment under Dark Conditions

Twenty-day-old *Arabidopsis* plants were transferred to a sound-proof chamber and subjected to 500 Hz at 80 dB (adjusted manually) for four different time periods (10, 30 m, 1 and 2 h) including the 1 h treatment as analyzed previously (Ghosh et al., 2016). A 1 h treatment was included for further confirming the stringency of our results. Control plants were kept in a similar chamber without SV treatment. The sound-proof chamber was customized by Korea Scientific Technique Industry (Korea) according to Jeong et al. (2008). This sound-proof chamber was not equipped with a light source and temperature controller. Sound intensity of the growth room, as recorded by a sound intensity meter TES-1350A (Pusung, Korea), was noted to be 75 ± 2 dB. The sound intensity within the chamber was recorded to be 40 dB. The Adobe Audition version 3.0 software (USA) was used for generation of single frequency sound. After the SV treatment, rosette samples were harvested for quantitative real-time PCR (qRT-PCR) analysis.

### SV Treatment under Lighted Condition

**FIGURE 1:**
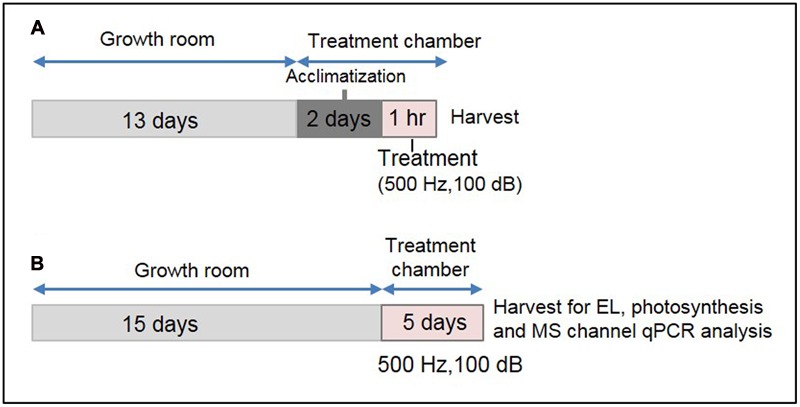
**Experimental set-up for SV treatment under lighted condition and the sample harvest strategy. (A)** Schematic representation of 1 h SV treatment. Thirteen-day-old plants were acclimatized in the specialized plant growth chamber. Five-hundred hertz (Hz) was applied to *Arabidopsis* for 1 h with 100 decibel (dB) sound intensity. Two separate experiments were carried out with chamber swapping and whole experiment was carried under continuous light condition. **(B)** Schematic representation of SV treatment for continuous 5 days. Fifteen-day-old plants were exposed to 500 Hz at 100 dB. After 5 days samples were harvested for electrolyte leakage (EL), photosynthetic parameter and MS ion channel qRT-PCR analyses.

Thirteen-day-old plants were covered with a transparent plastic dome to protect them from mechanical perturbation caused by direct air flow within the plant growth chamber. Two sides of the dome were cut manually to make a big hole, and placed perpendicularly to the air flow channel. Additionally, eight small holes were made on the top of the dome for gentle aeration. Plants covered by the dome were placed on a thick sponge cushion within the chamber to reduce the effect of mechanical vibration of the compressor. This set-up was maintained for 2 days under continuous light (∼150 ± 10 μmol m^-2^ s^-1^) at 22 ± 1°C within the growth chamber for the plants to get acclimatized. The plant growth chamber was equipped with a speaker (Sammi, Korea) and provision for watering from outside. A schematic diagram of this specialized plant growth chamber has been shown in Supplementary Figure S1. After the acclimatization, 1 h SV treatment (500 Hz, 100 ± 1 dB) was given under continuous illumination. The intensity of background noise within the chamber was 82 ± 2 dB. Control plants were acclimatized under similar condition without SV treatment. For long-term SV treatment, 15-day-old plants were transferred to this specialized plant growth chamber and treated continuously for 5 days. Plants were regularly watered upto 18 days. After 5 days, rosette leaves from 20-day-old SV-treated plants were harvested and used for the expression analysis of MS ion channel genes, EL and photosynthesis. Two independent experiments were performed by swapping the chambers between control and treatment (indicated as set 1 and 2 in the figures) to normalize the chamber effects and add more precision in our data. For a better representation of treatment method, a schematic diagram has been shown in **Figure 1**.

### Touch-Mediated Expression Profiling of SRGs and MS Ion Channel Genes

Gentle touch treatment was given to the 18-day-old plants in the growth room, as mentioned previously (Lee et al., 2005); 3–4 mature rosette leaves per plant were gently bent back and forth (four times) manually, and the samples (all touched and untouched leaves in a rosette) were harvested at 5, 15, 30 m and 1 h after the touch treatments. The control sample was harvested before the touch treatments. For long-term treatments, the 15-day-old plants were repeatedly touched (twice per day at 10 h interval) for 5 days. After 5 days, rosette leaves (both touched and untouched) from 20-day-old touch-treated plants were harvested and used for the expression analysis of MS ion channel genes. At the same time, 20-day-old untouched plants were harvested as control sample. Two independent experiments with long-term touch treatments were performed and results for both have been shown (indicated as set 1 and 2 in the figures).

### Developmental Stage-Specific Samples Collection

Rosette leaves (two types, young and mature), cauline leaves, young flowers (green and unopened buds) and mature flowers (white and fully opened), were harvested from 23-day-old plants. Samples from four different developmental stages (root, stem, young green pods and matured green pods) were harvested from 35-day-old plants. Ripening pods (fully developed and yellowish green in color) were collected from 38-day-old plants. Fifty to 100 seeds were washed twice, followed by soaking in water, and kept at 4°C for 2 days in dark. Imbibed seeds were subsequently transferred to the growth room under light for 10 h before harvesting. For the seedling sample, seeds were surface-sterilized and placed on plates containing 1× Murashige and Skoog (MS) salts and 0.4% phytagel (Sigma, USA), pH5.8. Subsequently, plates were kept at 4°C for 2 days in dark before transferring them to the growth room. Seeds were allowed to germinate, and 5-day-old seedlings were harvested.

### Quantitative Real-Time PCR (qRT-PCR) Analysis

Total RNA from an *Arabidopsis* rosette was extracted with the RNeasy Plant Mini kit (Qiagen, USA) and treated with DNase I (Qiagen) according to the manufacturer’s instruction. cDNA was prepared with 1 μg RNA, using GoScript Reverse Transcription system (Promega, USA), as per manufacturer’s instructions. cDNA was diluted 10-fold before using as a template for qRT-PCR analysis. qRT-PCR was performed using Mx3000P qPCR system (Agilent, USA) and LF Taq qPCR SYBR Mix (LPS Solution, Korea). Primer details of SRGs are available in Ghosh et al. (2016). Primer details of MS ion channels are available in Supplementary Table S1. At1g13440 (*GAPDH*) gene encoding glyceraldehyde-3-phosphate dehydrogenase was used as an internal control. We noticed the low coefficient of variation of *GAPDH* across SV treatments with various Hz at constant amplitude (Supplementary Table S2), which indicates relatively stable expression levels. Furthermore, Duncan’s multiple range test also indicated that there was no significant difference between C_T_ values of *GAPDH* in different Hz treatment at *P*-value 0.05. C_T_ values for all genes of interest (C_T_._GOI_) were normalized to the C_T_ values of *GAPDH* (C_T_._GAPDH_) for each replication [ΔC_T_ = (C_T_._GOI_) – (C_T_. _GAPDH_)] as suggested previously (Schmittgen and Livak, 2008). Relative transcript levels of each gene were calculated with respect to *GAPDH* (% relative expression to *GAPDH*) using 2^-ΔCT^ value [2^-ΔCT^ × 100] and plotted in the graph (Schmittgen and Livak, 2008). To isolate RNA from other developmental stages, a similar protocol was followed. RNA from imbibed seeds was isolated using the cetyltrimethylammonium bromide (CTAB) method as mentioned previously (Ghosh et al., 2012). Mean values and standard errors were obtained from four biological replicates.

### Measurement of Relative EL

Relative EL was measured as previously described by Cao et al. (2007), with slight modifications. Three to five matured rosette leaves per plants were cut, weighed (∼0.1 g) and washed with deionized water. Cut leaves were fully submerged in 20 ml of deionized water and shaken gently in a shaker incubator for 6 h (at 23°C, 140 rpm). Subsequently, the electrical conductivity (C1) was measured by a conductivity meter (Cond 3110, Incli.Teta Con@ 325, 2CA101, Germany). Later, the samples were boiled for 15 m in a water bath, following which the samples were allowed to cool to normal temperature. Conductivity was measured again for the second time (C2). Finally, relative EL was calculated using C1/C2.

### Measurement of Photosynthetic Parameters

Photosynthetic parameters were measured as previously described by Gururani et al. (2015). The maximum quantum of yield of photosystem II photochemistry (*Fv*/*Fm*) and performance index (PI) were measured using a Pocket PEA chlorophyll fluorometer (Hansatech, UK) in darkness-adapted plants. Furthermore, the chlorophyll-*a* fluorescence transients recorded in the darkness-adapted control and SV-treated *Arabidopsis* plants were analyzed by the so-called JIP-test (Strasser and Tsimilli-Michael, 2001) to study their structural and functional parameters that indirectly quantify the photosynthetic behavior of the experimental plants. The data are represented in the form of a radar plot which exhibits the calculated average values of the photosynthetic parameters of the control and SV-treated *Arabidopsis* plants. The measurements were taken with 5–7 matured rosette leaves per plants, and the average and standard error of means were calculated from 12 individual plants.

## Results

### Expression Pattern of SRGs after SV Treatment for Various Time Periods

**FIGURE 2:**
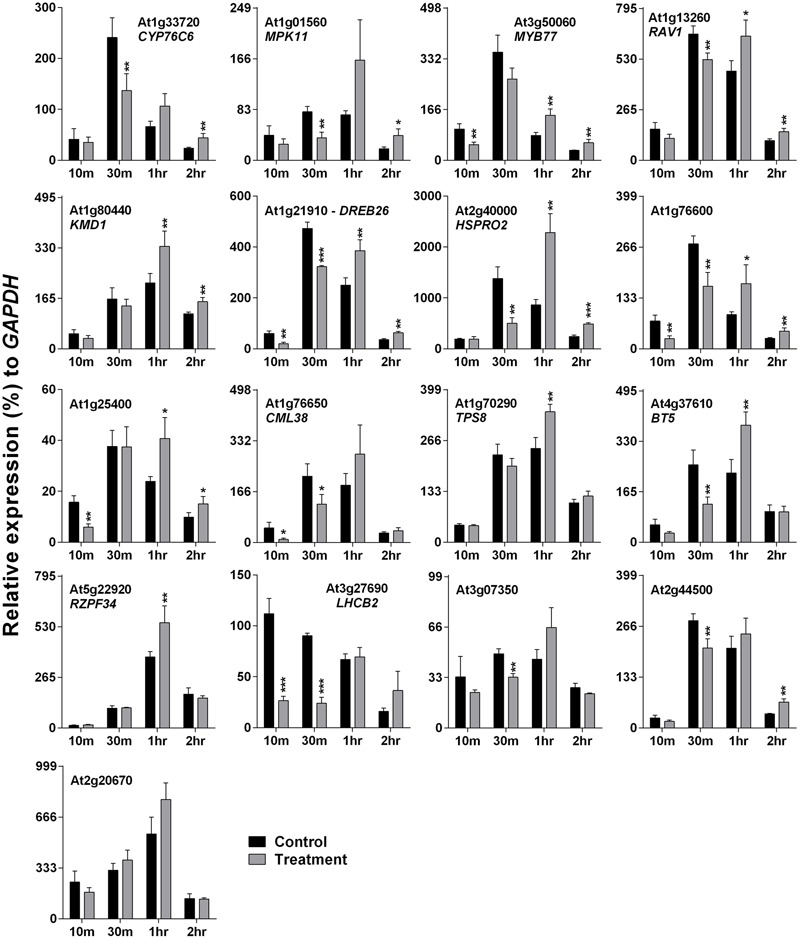
**Quantitative real-time PCR analysis of SV-regulated genes after exposure to 500 Hz for various time periods.** Expression of each gene in the *Arabidopsis* exposed to SV (gray) was compared with control (black). The time (10, 30 m, 1 and 2 h) indicates the duration of SV exposure in the sound-proof chamber. Intensity of the 500 Hz SV was manually set to 80 dB. Error bar indicates the standard error of means from four biological replications. *P*-value ranges are marked by asterisks: ^∗∗∗^*P* < 0.01, ^∗∗^0.01 < *P* < 0.05, ^∗^*P* < 0.1.

*Arabidopsis* plants were treated with 500 Hz SV for four different time periods: 10, 30 m, 1 and 2 h. Plants were exposed to SV under darkness in a specialized sound-proof chamber, and control plants were kept in a similar chamber without SV. Broadly, the SRGs were down-regulated after short exposures (10 and 30 m), and up-regulated after long exposures (1 and 2 h) of SV (**Figure 2**). The overall expression pattern was bell-shaped: started with low expression at 10 m, followed by highest expression at 30 m or 1 h, and reduced again at 2 h. On the basis of the up-regulation pattern, we can categorize genes into two groups: (1) genes which were up-regulated either at 1 or 2 h (*CML38, TPS8, BT5, RZPF34, LHCB2, At3g07350, At2g44500* and *At2g20670*), and (2) the ones which were up-regulated at both time points (*CYP76C6, MPK11, MYB77, RAV1, KMD1, DREB26, HSPRO2, At1g76600* and *At1g25400*).

### Cross-Confirmation of SV-Regulated Genes’ Expression under Light Condition

**FIGURE 3:**
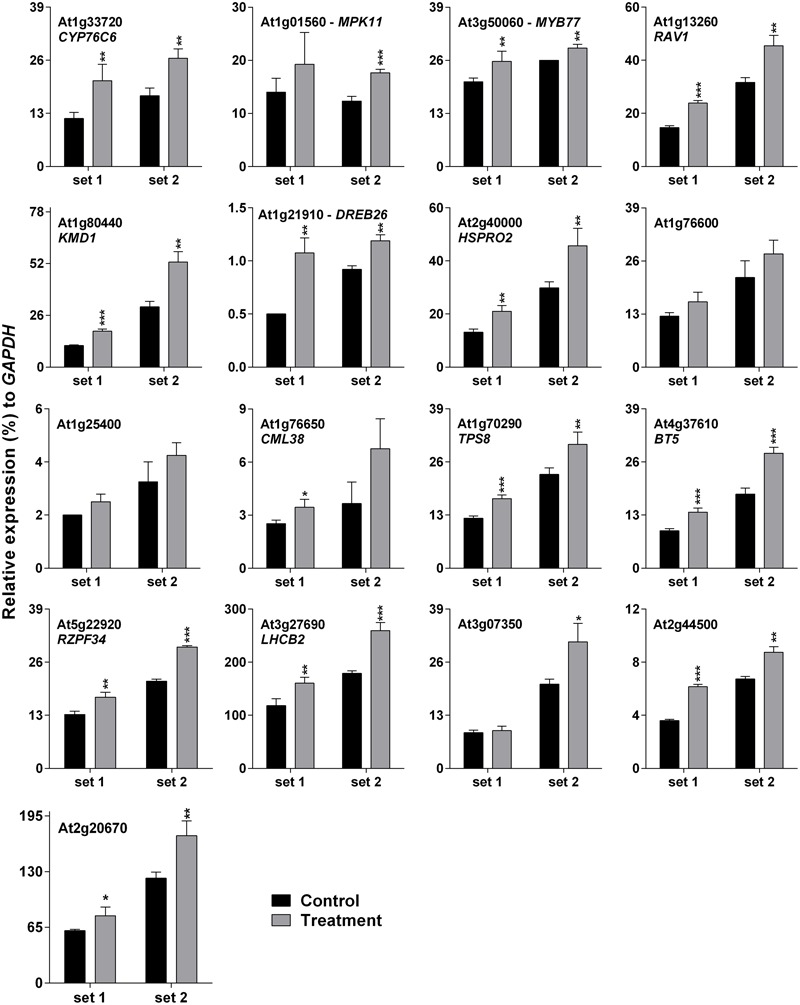
**Quantitative real-time PCR analysis of SV-regulated genes under lighted condition.** Expression of each gene in the *Arabidopsis* exposed to a 500 Hz SV for 1 h (gray) was compared with control (black). Intensity of the 500 Hz SV was 100 dB. Set 1 and 2 indicate the two independent experiments with chamber swapping between control and treatment. Error bar indicates the standard error of means from four biological replications. *P*-value ranges are marked by asterisks: ^∗∗∗^*P* < 0.01, ^∗∗^0.01 < *P* < 0.05, ^∗^*P* < 0.1.

Darkness-mediated up-regulation of 14 out of 17 genes was noted previously (Lee et al., 2005), which is summarized in Supplementary Table S3. Cross-talk between light/dark transition and SV treatment could be the reason for initial down-regulation (at 10 and 30 m) of SRGs (**Figure 2**). Therefore it was necessary to confirm SRGs’ expression under lighted condition. For this purpose, plants were exposed to 500 Hz for 1 h under lighted condition in a specialized plant growth chamber (**Figure 1A**). Similarly, to reduce the effect of movement of plants from growth room to chamber, we acclimatized the plants in the chamber for 2 days prior to SV exposure (**Figure 1A**). Experiments under lighted condition also confirmed the SV-mediated induction of these genes (**Figure 3**). Besides that, repeating the experiments with chamber-swapping conditions (marked as set 1 and 2) also confirmed the reproducibility of the set-up for future research.

### Touch-Mediated Expression Profiling of SRGs

**FIGURE 4:**
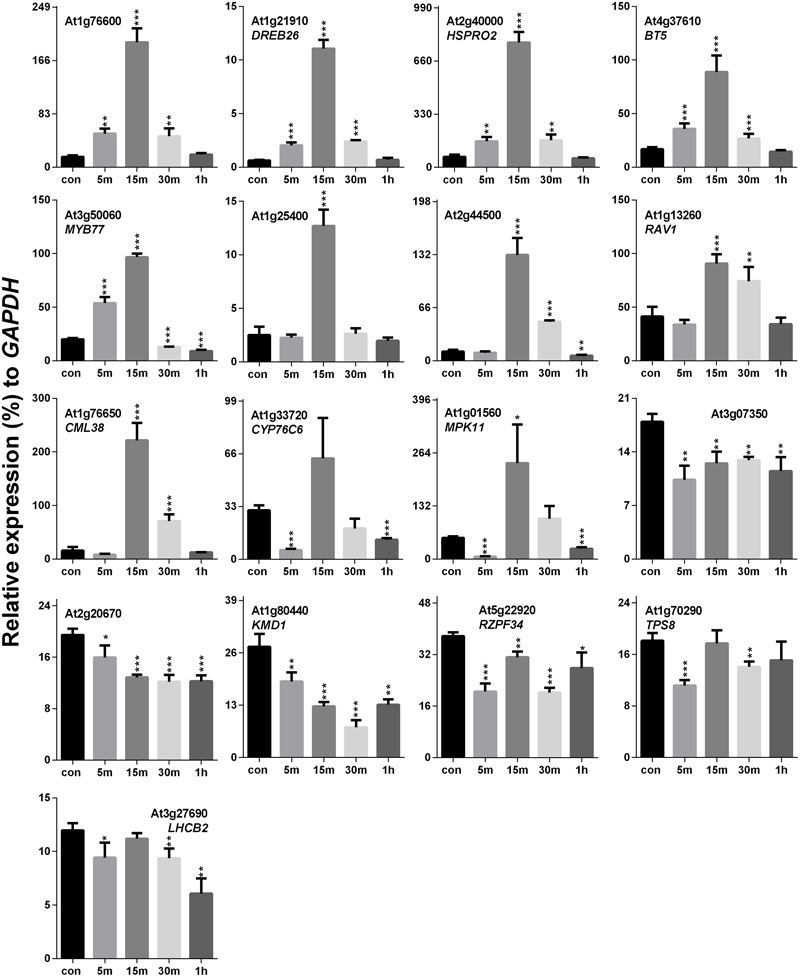
**Quantitative real-time PCR analysis of SV-regulated genes after touch treatment.** The time (5, 15, 30 m and 1 h) indicates sample harvesting time after touch treatment. The control sample (black) was harvested before the touch treatment. Error bar indicates the standard error of means from four biological replications. *P*-value ranges are marked by asterisks: ^∗∗∗^*P* < 0.01, ^∗∗^0.01 < *P* < 0.05, ^∗^*P* < 0.1.

Previously, it was hypothesized that SV and touch share some common mechanosensitive (MS) signaling events (Ghosh et al., 2016). Therefore, touch-mediated expression profiling of SRGs is necessary to elucidate their similarities and/or dissimilarities at the molecular level. Touch treatment resulted in up- and down-regulation of 11 and 4 genes, respectively, out of the 17 SRGs (**Figure 4**). On the basis of the expression pattern, we categorized these genes into four distinct groups: (1) genes that were highly up-regulated at 15 m (eight in no; *At1g76600, DREB26, HSPRO2, BT5, MYB77, At1g25400, At2g44500* and *RAV1)*, (2) those which were down-regulated at 5 m and 1 h, but up-regulated at other time points (*CML38, CYP76C6* and *MPK11*), (3) the ones down-regulated at each time point (*At3g07350, At2g20670, KMD1* and *RZPF34*), and (4) genes without any specific trend, but down-regulated at some time points (*TPS8* and *LHCB2*).

### Developmental Stage-Specific Expression Pattern of SRGs

Thirteen different samples were analyzed by qRT-PCR, which are as follows: root, stem, leaves (four types; cauline, young, mature, and senescence), flowers (two types; young and mature), pods (three types; young, mature, and ripening), imbibed seeds and seedlings (Supplementary Figure S2; Supplementary Table S4). On the basis of high expression pattern, we categorized these genes broadly into four groups: (1) genes that were restricted in one developmental stage (*MYB77* in imbibed seed and *At3g07350* in seedlings), (2) genes that were restricted in one tissue type (*LHCB2* and *CYP76C6* in leaves), (3) highly expressed genes in two to three developmental stages (*CML38, HSPRO2* in imbibed seeds and senescing leaves; *KMD1* in seedlings and senescing leaves; *At2g20670, DREB26* in seedlings and leaves; *At1g25400* in young and senescing leaves; *TPS8* in imbibed seeds, seedlings and senescing leaves; *At1g76600* in imbibed seeds, mature leaves and senescing leaves) and (4) highly expressed genes in more than three developmental stages (*At2g44500, RZPF34, MPK11, RAV1*, and *BT5*). However, statistically highest expression of some genes from group 2 to 4 were observed in the following developmental stages: imbibed seeds (*CML38, HSPRO2* and *TPS8*), seedlings (*KMD1, At2g20670, DREB26, RZPF34*, and *BT5*), young leaves (*CYP76C6, LHCB2, DREB26* and *RAV1*) and senescing leaves (*At1g25400, At1g76600*, and *MPK11*).

### Assessment of Changes in EL and Photosynthetic Machinery in the SV-Treated Plants

**FIGURE 5:**
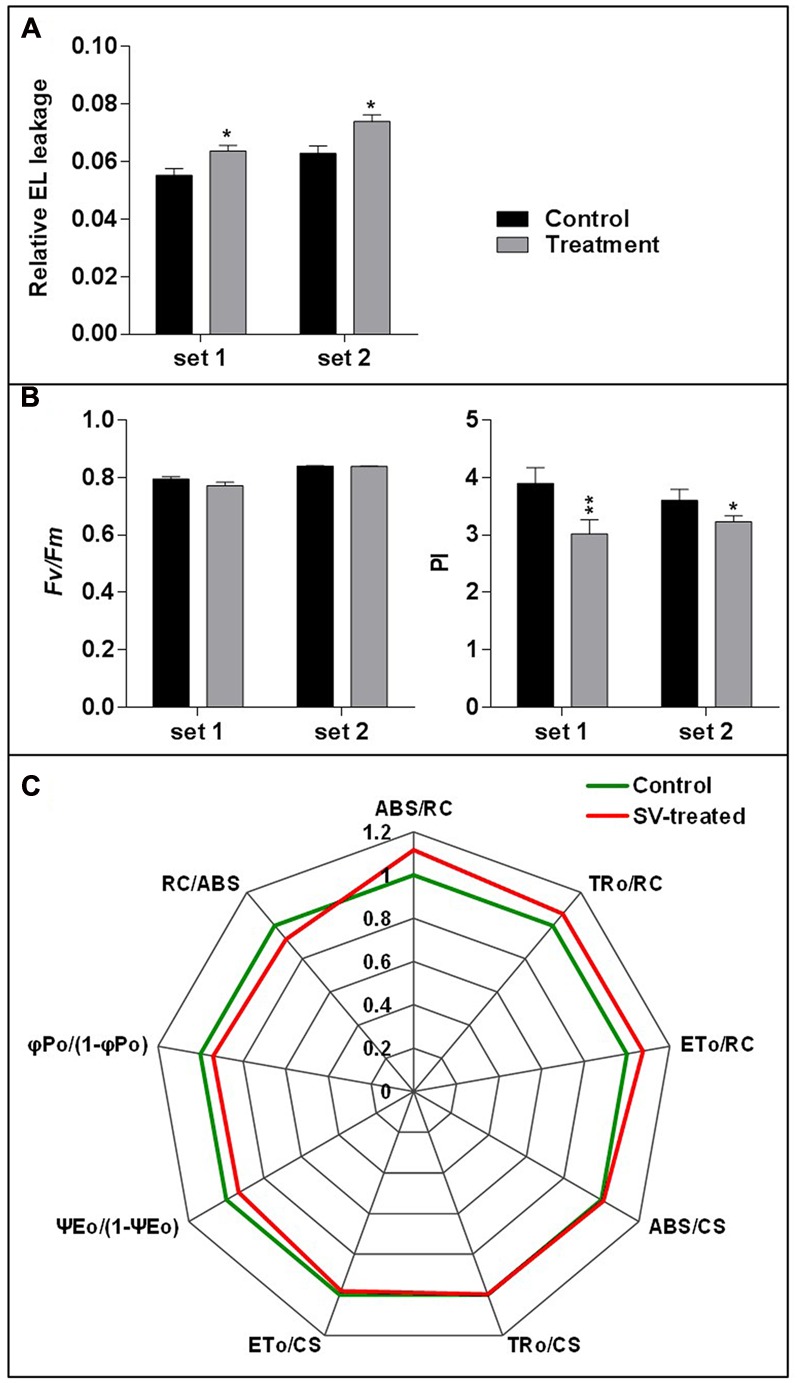
**Measurement of relative electrolyte leakage and photosynthetic parameters. (A)** The relative electrolyte leakage of leaves was measured after 5 days of SV treatment (*n* = 10). **(B)** Similarly, maximum quantum yield of photosystem II photochemistry (*Fv*/*Fm*) and performance index (PI) were measured after 5 days of SV treatment (*n* = 12). Black and gray colors indicate control and SV-treated plants, respectively. Set 1 and 2 indicate the two independent experiments with chamber swapping. Error bar indicates the standard error of means. Asterisks indicate the level of significance as determined by *t*-test (^∗∗^0.01 < *P* < 0.05, ^∗^*P* < 0.1). **(C)** Radar plot with a series of parameters derived from JIP-test analyses of the fast OJIP transients exhibiting the differences in the structure and function of the photosynthetic apparatus in control (green) and SV-treated (red) *Arabidopsis* plants. The OJIP-test used in this study, delineates the maximal energy fluxes for the following photosynthetic events- absorption (ABS), trapping (TRo), and electron transport (ETo) in the energy cascade quantified by the fluxes per cross section (CS) and per reaction center (RC) in SV-treated plants compared to those of control plants. Any marginal difference in these parameters between SV-treated and control plants is seen as a change in primary photochemistry of PSII. ABS/RC, light absorption flux (for PSII antenna chlorophylls) per RC; TR_0_/RC, trapped (maximum) energy flux (leading to Q_A_ reduction) per RC; ET_0_/RC, maximum electron transport flux (further than Q_A_-) per PSII RC; ABS/CS, absorbance of photons per excited CS, TR_0_/CS, trapped energy flux per excited CS; ET_0_/CS, maximum electron transport flux (further than Q_A_-) per excited CS; ψEo, efficiency/probability for electron transport (ET), i.e., efficiency/probability that an electron moves further than Q_A_- at *t* = 0; φPo, maximum quantum yield for primary photochemistry at *t* = 0; RC/ABS, density of reaction centers per PSII antenna chlorophyll. Data are mean ± SE (*n* = 4, *P*-value for comparison of treatments: <0.05).

Electrolyte leakage and photosynthetic parameters were analyzed to check the SV-mediated physiological response after 5 days of long exposure. EL is a measurement for the ionic loss through the plasma membrane caused by external stimuli (Cao et al., 2007). Higher EL was observed after 5 days of SV treatment compared to control (**Figure 5A**). Simultaneously, two photosynthetic parameters were measured: (i) *Fv/Fm*, which indicates the quantum efficiency of photosystem II and (ii) PI, which shows the PI for energy conservation from excitation to the reduction of PSI end acceptors. PI was significantly reduced after 5 days of SV treatment compared to control, though no significant change was observed in *Fv/Fm* (**Figure 5B**). The comparative diurnal behavior of nine critical biophysical PS II characteristics in control and SV-treated plants is presented as a radar plot in **Figure 5C**. All data were normalized to the reference (control plants) and each variable at reference was standardized by giving it a numeric value of 1. All variables were deduced from the JIP-test analysis. Any deviation in these parameters relative to their control values represents a change in the PSII efficiency and it has been used earlier in numerous studies (Smit et al., 2009; Gururani et al., 2015). The parameter ABS/RC indicates the total absorption of PSII antenna chlorophylls per active reactive center (RC), while ET_0_/RC reflects the transport of electrons to the photosynthetic electron transport chain per active PSII-RC. The trapping of an excited photon by the RCs leads to the reduction of quinone, and since at time zero, the RCs are considered open, the maximal trapping of excited photons per RC can be represented as TR_0_/RC. The increased value of ABS/RC, ET_0_/RC and TR_0_/RC in SV-treated plants suggested that the absorption and trapping flux of photons, as well as the electron transport per active RCs was higher in these plants compared to those in control plants. Notably, a concomitant increase in ABS/RC is seen as an increase in the apparent size of the antenna rather than a structural increase in the antenna size of a biochemical complex (Strasser et al., 2004; Smit et al., 2009; Redillas et al., 2011). Next, the phenomenological energy fluxes per excited cross-section (CS) for absorption (ABS/CS), trapping (TRo/CS) and electron transport (ETo/CS) were calculated. We found that the amount of chlorophyll per leaf area of the tested samples was either similar or marginally higher than the control plants after 5 days of SV treatment. The parameter ΨE_0_/(1 – ΨE_0_) which indicates the efficiency of a trapped exciton to transport an electron into the photosynthetic electron transport chain was recorded to be lower in SV-treated plants than in control plants. Reduced values of ΨE_0_/(1 – ΨE_0_) with increasing salt concentration in wheat plants and in cold-stressed turf grasses have been reported earlier (Mehta et al., 2010; Gururani et al., 2016). Similarly, the maximum yield of primary photochemistry φPo/(1-φPo) where φPo = TRo/ABS was also recorded to be lower in SV-treated plants compared to that in control plants, SV treatment negatively influenced these parameters after 5 days of SV treatment. This could perhaps be corroborated with the reduced PI values in SV-treated plants (**Figure 5B**). PI is an indirect indicator of the vitality of plant samples where a reduced vitality is expressed in terms of reduced PI values. However, earlier studies have indicated that since electron flux is used in both carbon metabolism and other biochemical pathways, PI values are only indirectly related to the net photosynthesis (Bussotti et al., 2007). PI essentially includes three photosynthetic parameters: (1) the density of reaction centers (RC/ABS); (2) the quantum yield of primary photochemistry of PSII (φPo = TRo/RC), and (3) ability to feed electrons into the photosynthetic electron transport chain between PSII and PSI (Ψ_0_ = ETo/TRo) (Strasser and Tsimilli-Michael, 2001; Bussotti et al., 2007).

### Expression Analysis of MS Ion Channels after SV and Touch Treatment

**FIGURE 6:**
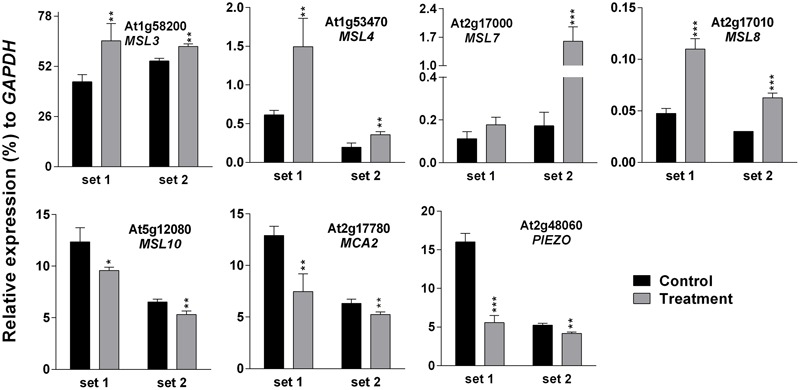
**Quantitative real-time PCR analysis of MS ion channel genes after exposure to SV.** Plants were exposed to 500 Hz SV with 100 dB intensity for 5 days in a specialized plant growth chamber. Expression of each gene in the *Arabidopsis* exposed to SV (gray) was compared with control (black). Set 1 and 2 indicate the two independent experiments with chamber swapping between control and treatment. Error bar indicates the standard error of means from four biological replications. *P*-value ranges are marked by asterisks: ^∗∗∗^*P* < 0.01, ^∗∗^0.01 < *P* < 0.05, ^∗^*P* < 0.1.

**FIGURE 7:**
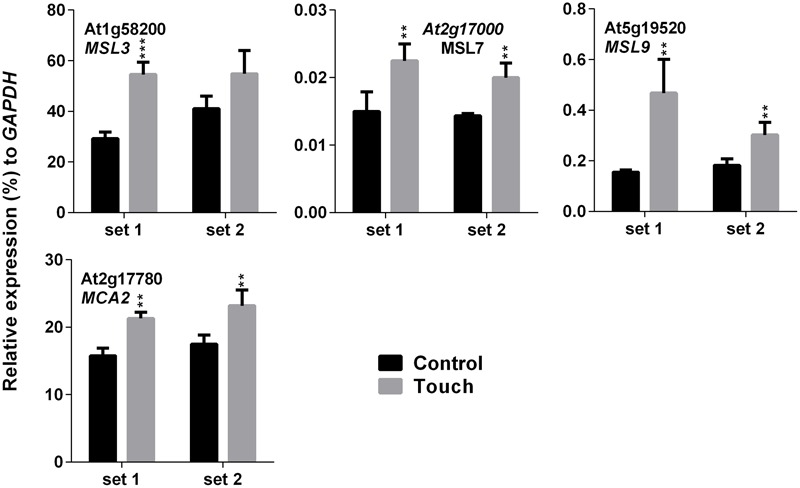
**Quantitative real-time PCR analysis of MS ion channel genes after repetitive touch treatments.** Plants were repeatedly touched for 5 days. Expression of each gene in the *Arabidopsis* treated with touch (gray) was compared with control (black). Set 1 and 2 indicate the two independent experiments. Error bar indicates the standard error of means from four biological replications. *P*-value ranges are marked by asterisks: ^∗∗∗^*P* < 0.01, ^∗∗^0.01 < *P* < 0.05.

Higher EL in SV-treated plants can be caused by the modulation of membrane integrity or alteration of channel activity. Therefore, expression analysis of MS ion channels after SV treatment could be interesting. Herein we investigated the expression pattern of 13 MS ion channels after 5 days of SV treatment which are as follows: *MSLs* (1–10), *MCAs* (1 and 2) and *Piezo*. Among them, seven genes had a similar expression pattern between chamber swapping experiments. Four genes (*MSL3, MSL4, MSL7* and *MSL8*) were up-regulated, and three genes (*MSL10, MCA2* and *PIEZO*) were down-regulated in the SV-treated plant (**Figure 6**). Simultaneously, we checked the expression of MS ion channels after touch treatment for comparing two mechanostimuli: SV and touch. Four genes (*MSL3, MSL7, MSL9* and *MCA2*) were up-regulated by 5 days of touch treatments in two independent experiments (**Figure 7**). Among them, *MSL3* and *MSL7* had similar up-regulation patterns between touch treatment and SV treatment, but *MCA2* showed an opposite expression pattern: down-regulated after SV treatment and up-regulated after touch treatment. The rest of the genes which showed inconsistent results between two sets of independent experiments after mechanical stimulation have been shown in Supplementary Figure S3.

## Discussion

The expression pattern of SRGs after exposure to SV for four different time periods in darkness was revealed (**Figure 2**). Up-regulation was observed after long exposure (1 and 2 h) of SV, though the magnitude of expression was reduced at 2 h. Surprisingly, the short exposure (10 and 30 m) of SV down-regulated the genes compared to the control. Previously strong correlation between darkness and touch treatments at transcript level was reported (Lee et al., 2005). More than 50% of touch- and darkness-induced genes were common (Lee et al., 2005). On the basis of our previous report as well, many up-regulated genes were common between touch and SV treatments (Ghosh et al., 2016). Among the SRGs, 14 were already noted to be up-regulated by 30 m of darkness, which are marked in Supplementary Table S3 (Lee et al., 2005). Besides that, nine genes (*MYB77, DREB26, HSPRO2, RAV1, MPK11, At3g07350, At2g44500, At1g76600* and *At1g25400*) were common in touch- and SV-mediated induction (Supplementary Table S3). It is reported that both light/dark transition and touch treatments are capable of altering membrane potential in plants (Koselski et al., 2008; Chehab et al., 2011). Besides, we hypothesized that both touch and SV have a common mechanical impact in general (Ghosh et al., 2016). Previously, slight difference in transcript levels of fructose 1, 6-bisphosphate aldolase (*ald*) and rubisco small sub-unit (*rbcS*) genes were noticed after the SV treatment between light- and dark- grown rice plant (Jeong et al., 2008). Collectively, our result generates a strong notion of molecular cross-talk between touch, SV and light/dark stimuli. In the future, a detailed study is needed to establish a strong correlation between these three stimuli.

The sudden transfer of light-grown plants to darkness and exposure to SV could create antagonistic molecular events. Consequently, dark and SV treatment together resulted in initial down-regulation (at 10 and 30 m) of SRGs as compared to the non-competitive dark effect in the control plants (**Figure 2**). Nevertheless, SV exposure for a long time overcame the dark acclimatization and resulted in eventual induction of the SRGs. Simultaneously, movement of plants from growth room to the sound-proof chambers may provoke molecular alteration in plants (**Figure 2**). Hence, it was necessary to confirm the expression of these genes in acclimatized plants by exposure to SV under lighted condition. Swapping experiments in the illuminated chamber confirmed the SV-induced modulation of these genes (**Figure 3**). Additionally, the light-equipped SV treatment chamber proved to be useful for long-term SV treatment in future research. Of course, a gene transcription can be modulated by multiple environmental stimuli. Correlating a gene’s expression with a particular stimulus definitely needs rigorous study at the molecular level. Therefore, mechanistic investigation in future may shed more lights on the SV-mediated regulation of these genes.

The majority of the SRGs were expressed spatiotemporally (Supplementary Figure S2). Among them, various genes are involved in plant growth and development. SV-mediated plant growth promotion was observed previously (Hassanien et al., 2014). These genes might have a role in SV-mediated plant growth promotion. KMD1 and DREB26 encode a kelch repeat containing F-box protein and an AP2/ERF family protein, respectively. BT5 protein contains BTB and TAZ domain together. Several members of F-box, BT and AP2/ERF family are involved in the various processes of plant development (Kuroda et al., 2002; Robert et al., 2009; Krishnaswamy et al., 2011). CYP76C6 is a member of the cytochrome P450 subfamily C. P450s are involved in the phytohormone metabolic process and regulate plant growth and development (Xu et al., 2015). RAV1, an AP2/EREBP (APETALA2) type of TF involves in the flowering and senescence processes (Matias-Hernandez et al., 2014). Besides plant growth promotion, SV can induce the process of seed germination as well (Hassanien et al., 2014). The genes which were highly expressed in imbibed seeds probably have roles in SV-mediated seed germination. MYB77 is believed to interact with auxin response factors (ARFs) and involve in auxin response (Shin et al., 2007). Auxin is a positive regulator of gibberellic acid (GA) response and biosynthesis (Weiss and Ori, 2007); as GA is an important hormone for seed germination, this may be a reason behind SV-mediated enhancement in seed germination. Ca^2+^ fluxes are also considered to be crucial players during the germination process (Duval et al., 2002). Ca^2+^ binding calmodulin (CaM) has a role in sequestering cellular Ca^2+^ to maintain physiological range and avoid cytotoxicity (Duval et al., 2002). Induction of CaM transcripts was observed in pea during seed imbibition and germination (Duval et al., 2002). Therefore, it could be assumed that CaM-like (*CML38)* protein might be involved in similar processes. TPS enzyme converts glucose-6-P to trehalose-6-P, a positive regulator of seed germination (Tsai and Gazzarrini, 2014). Therefore, TPS8 might also be involved in the germination process. Direct correlation of these genes with SV-mediated germination or the developmental process needs detailed study in the future.

To check the effect of long exposure on a plant’s physiology, 500 Hz at 100 dB was applied for 5 days in the light-equipped SV treatment chamber and the EL and photosynthetic parameters were checked (**Figure 5**). Changes in molecular events such as gene expression and changes in photosynthetic efficiency in plants should not be seen as isolated events. Hence, an indirect approach called chlorophyll-a fluorescence analysis was employed to determine changes in the photosynthetic efficiency in SV-treated plants. Data from these studies indicated a significant change in the photosynthetic machinery after the plants were exposed to SV treatment for 5 days (**Figures 5B,C**). Previously, chlorophyll-a fluorescence studies have largely been used to assess the photosynthetic performance and overall physiology of plants under abiotic stresses (Gururani et al., 2013, 2016; Zurek et al., 2014). On the basis of the present data it can be concluded that this indirect approach of evaluating photosynthetic efficiency of plants can also be used in SV-treated plants. Measurement of EL helps to understand the status of plasma membrane integrity, which can be easily affected by external stressors (Cao et al., 2007). Previous studies have shown that SV can alter cell wall and membrane microstructure which leads to a change in tension of the cell membrane (Bochu et al., 2001; Mishra et al., 2016). SV-mediated changes in lipid fluidity and protein secondary structure of plasma membrane were observed in chrysanthemum and tobacco, respectively (Zhao et al., 2002b; Yi et al., 2003). Therefore, it could be assumed that being a pressure wave, SV exerted pressure on cell wall- plasma membrane microstructure and altered its integrity which resulted in higher EL compared to the control plant. Additionally, SV-mediated changes in the activity of membrane-associated channels may alter the EL. It was previously noted that SV can alter K^+^ channel permeability and H^+^-ATPase activity in chrysanthemum (Zhao et al., 2002a,c). It was hypothesized that MS ion channels might be involved in the perception of the mechanical signals (Monshausen and Haswell, 2013). The role of MS ion channels for touch sensing was observed in earlier studies (Shepherd et al., 2002; Nakagawa et al., 2007). To check whether SV has any effect on the transcript level of MS ion channels, herein we investigated their expression pattern. The result showed that SV treatment continuously for 5 days up-regulated four genes (*MSL3, MSL4, MSL7*, and *MSL8*) and down-regulated three genes (*MSL10, MCA2* and *PIEZO*) (**Figure 6**). SV-mediated differential expression of channel genes may be involved in the higher EL, and triggers the downstream signaling processes.

Touch is considered as an external mechanical force like SV. Touch-mediated gene induction pattern was broadly spiking in nature, and was normalized to control level after 1 h (**Figure 4**). Hence, time points for expression analysis after touch treatments are crucial. Surprisingly, six SRGs (*At3g07350, At2g20670, KMD1, RZPF34, TPS8* and *LHCB2*) were not up-regulated by touch at any of the time points. On the other hand, *MCA2* showed the opposite expression pattern after 5 days of SV and touch treatments, i.e., down-regulated after SV treatment and up-regulated after touch (**Figures 6** and **7**). This variation indicates the difference between two mechanostimuli at molecular level. Therefore, these seven genes could be the interesting candidates to highlight the differences between touch- and SV-mediated molecular responses in future. It has already been noted that the expression of *MSL8* and *MSL9* are mainly restricted in *Arabidopsis* flower and root, respectively (Haswell et al., 2008; Hamilton et al., 2015). In corroboration with this, we also observed very low expression of these two genes compared to other ubiquitously expressed MS ion channel genes (like- *MSL2, MSL3, MSL5, MSL6, MSL10, MCA1* and *MCA2*). A detailed tissue-specific analysis of MS-ion channel genes after mechanical stimulation can lead to more interesting inferences in future.

## Conclusion

SRGs could be regulated by darkness and touch treatments. Both being mechanostimuli, touch and SV may share some common MS signaling events. Besides, the distinct expression pattern of six SRGs and *MCA2* generates an idea that SV is perceived as a stimulus distinct from touch. Certainly, a detailed comparative study is required to elucidate the similarities and dissimilarities between these two mechanostimuli. Additionally, the spatiotemporal expression of SRGs could be linked to SV-mediated growth promotion and germination by extensive research in the future. Induction of chemical defense by chewing sound of caterpillar in *Arabidopsis* and elevated level of polyamine by natural sound in Chinese cabbage (Qin et al., 2003; Appel and Cocroft, 2014) indicate the ecological and/or environmental significance of SV to plants. Corroboratively, ‘Buzz Pollination,’ a phenomenon where pollen from anthers are released at a particular frequency produced by bee’s buzz (De Luca and Vallejo-Marin, 2013), also highlights the environmental significance of SV. Such kind of natural SVs can be the interesting stimulus for extending the plant-acoustic research to molecular level. Therefore, comparative investigation with various ecologically significant SVs in natural photoperiod is required to give impetus to this least explored area of plant science.

## Author Contributions

RG performed most of the experiments. LP performed the experiment shown in **Figure 6**. RG and RM analyzed the data. MG analyzed the data shown in **Figure 5C**. RG, S-CP, M-JJ, and HB conceived the idea and designed the experiments. HB supervised the experiments. RG, MG, RM, and HB wrote the manuscript. All the authors approved the final manuscript.

## Conflict of Interest Statement

The authors declare that the research was conducted in the absence of any commercial or financial relationships that could be construed as a potential conflict of interest.
