# Supplementary material for: Expression Analysis of Sound Vibration-Regulated Genes by Touch Treatment in Arabidopsis
Source: Front Plant Sci. 2017 Jan 31;8:100. doi: 10.3389/fpls.2017.00100 (PMC5281610; doi:10.3389/fpls.2017.00100)

## *Supplementary Material*

## Expression Analysis of Sound Vibration-Regulated Genes by Touch Treatments in Arabidopsis.

Ritesh Ghosh^1^, Mayank Anand Gururani^2^, Lakshmi Narayanan Ponpandian^1^, Ratnesh Chandra Mishra^1^, Soo-Chul Park^3^, Mi-Jeong Jeong^3^, Hanhong Bae^1,^*

^1^ Department of Biotechnology, Yeungnam University, Gyeongsan 38541, Republic of Korea.

^2^ Department of Biology, College of Science, United Arab Emirates University, Al Ain 15551, United Arab Emirates.

^3^ National Institute of Agricultural Sciences, Rural Development Administration, Korea

(South)

**Correspondence:**

Hanhong Bae

[hanhongbae@ynu.ac.kr](mailto:hanhongbae@ynu.ac.kr)

**Supplementary Table S1.** List of primers for Quantitative real-time PCR analysis of MS ion channel genes.

| **Genes** | **Forward primer** | **Reverse primer** |
| --- | --- | --- |
| At4g00290  **MSL1** | GCTTGATAGGGAGAAGGTATTGACT | CTTCTACGGATCCAGCTTTGATAGT |
| At5g10490  **MSL2** | ACCAACAAAGTACCTGAAGAAACAG | AGTGTTCTCTTTGAGCCTTCTAGTG |
| At1g58200  **MSL3** | ACGAAGCTACAATAGAGCAGACACT | CAAGTTCTTCTGAATCCATTAGTGC |
| At1g53470  **MSL4** | GCTAACTATTACCGCAGTCCTGATA | TCTCCTCACATATCTCTCTCCCATA |
| At3g14810  **MSL5** | ATCATATATCCGAATAGTGTCCTCG | ACTATTCAAGTCATCCATGCTTAGG |
| At1g78610  **MSL6** | GTATCGAATTCTCCATCCACATTAC | ATCTCTTCAACAAGTTGAGACCTTC |
| At2g17000  **MSL7** | GTCCACATTACTACTCCTCCTGAAA | CAACGCTCTTCTTGTAAATCTCTCT |
| At2g17010  **MSL8** | GTGAGATTGACAGTGTACAGTTGGT | TGTCGATGTAGTTCGATATTCTCTG |
| At5g19520  **MSL9** | TTCTACAGAAGTCCAGATATGGGAG | GCGGTTCTTCTTAAACTCTTCTCTA |
| At5g12080  **MSL10** | TATCTCTTTCTCGACACCAGTCTCT | CGGTTCTTCTAAGATTCCTCTCTCT |
| At4g35920  **MCA1** | GATCCACTTCTAATGTTTCATCTGG | GTATCAAAGAATACGCCATTAGCTC |
| At2g17780  **MCA2** | CTTTGATACTCTCATGCTGTTGCTA | TGGTGGACTCATTTCCTTATTACTC |
| At2g48060  **PIEZO** | CAGTTGCTATCATAATGTCTGAGGA | CAAGCGTCCAGTATAGAACTTCTTC |

**Supplementary Table S2.** Validation of At1g13440 (*GAPDH*) gene encoding glyceraldehyde-3-phosphate dehydrogenase as internal control for qRT-PCR analysis. We noticed the low coefficient of variation of *GAPDH* across SV treatments with various Hz at constant amplitude, which indicates relatively stable expression levels. The below table indicates the C_T_ values of *GAPDH* across the given treatments.

| Treatments | 250 Hz | 500 Hz | 1000 Hz | 2000 Hz | 3000 Hz |
| --- | --- | --- | --- | --- | --- |
| Average C_T_ | 21.8 | 21.5 | 21.6 | 21.8 | 21.5 |
| Coefficient of variation (CV) | 0.046 | 0.044 | 0.037 | 0.033 | 0.035 |

**Supplementary Table S3.** List of SV-regulated genes showing their expression pattern in Arabidopsis after touch, dark and sound vibration treatments. Arrow mark indicates treatment-mediated up-regulation of the genes. Blank space indicates the genes which had marginal or undetectable expression on at least one of the nine chips during the analysis of touch- and darkness- mediated expression.

| **Locus** | **Gene** | **Touch** | **Dark** | **Sound** |
| --- | --- | --- | --- | --- |
| At1g33720 | CYP76C6 |  | ↑ | ↑ |
| At1g01560 | MPK11 | ↑ | ↑ | ↑ |
| At3g50060 | MYB77 | ↑ | ↑ | ↑ |
| At1g13260 | RAV1 | ↑ | ↑ | ↑ |
| At1g80440 | KMD1 |  | ↑ | ↑ |
| At1g21910 | DREB26 | ↑ | ↑ | ↑ |
| At2g40000 | HSPRO2 | ↑ | ↑ | ↑ |
| At1g76600 | -- | ↑ | ↑ | ↑ |
| At1g25400 | -- | ↑ | ↑ | ↑ |
| At1g76650 | CML38 |  |  | ↑ |
| At1g70290 | TPS8 |  | ↑ | ↑ |
| At4g37610 | BT5 |  |  | ↑ |
| At5g22920 | RZPF34 |  | ↑ | ↑ |
| At3g27690 | LHCB2 |  |  | ↑ |
| At3g07350 | -- | ↑ | ↑ | ↑ |
| At2g44500 | -- | ↑ | ↑ | ↑ |
| At2g20670 | -- |  | ↑ | ↑ |

**Supplementary Table S4.** Statistical analysis (Duncan’s multiple range test, DMRT) of SV-regulated genes in various developmental stages. The different letters indicate significant differences among each other with *P* < 0.05 (n=4) as determined by DMRT. Thirteen tissue samples were analyzed; root (R), stem (S), cauline leaves (CL), young leaves (YL), mature leaves (ML), senescent leaves (SL), young flower (YF), mature flower (MF), young pod (YP), mature pod (MP), ripening pod (RP), imbibed seed (IS) and seedling (slg).

|  | **At3g50060** | **At3g07350** | **At3g27690** | **At1g33720** | **At1g76650** | **At2g40000** | **At1g80440** | **At2g20670** | **At1g21910** | **At1g25400** | **At1g70290** | **At1g76600** | **At2g44500** | **At5g22920** | **At1g01560** | **At1g13260** | **At4g37610** |
| --- | --- | --- | --- | --- | --- | --- | --- | --- | --- | --- | --- | --- | --- | --- | --- | --- | --- |
| **root** | bc | b | d | e | bc | dc | dc | d | bc | bc | cd | bac | bdec | ed | c | bac | ab |
| **stem** | bc | b | d | de | c | d | c | d | c | bc | cd | bac | bdac | bedc | c | bac | bdc |
| **CL** | bc | b | b | b | c | dc | dc | cbd | ab | bc | cd | bac | fdec | bedc | ab | bac | bdc |
| **YL** | bc | b | a | a | bc | d | dc | bc | a | b | c | bc | bac | bdc | ab | a | bdc |
| **ML** | b | b | c | c | c | dc | dc | cbd | bc | c | d | ab | fbdec | ed | bc | bc | bdc |
| **SL** | bc | b | d | c | b | b | b | b | c | a | b | a | ab | bedc | a | ba | bc |
| **YF** | bc | b | d | de | c | d | d | cd | c | c | d | c | fe | bdc | c | d | bc |
| **MF** | bc | b | d | de | c | dc | dc | cd | c | bc | cd | c | a | ba | ab | d | bac |
| **YP** | bc | b | d | de | c | d | d | d | c | c | d | c | fbdec | bac | c | d | bdc |
| **MP** | bc | b | d | d | c | d | d | d | bc | c | d | c | fdec | edc | c | d | d |
| **RP** | bc | b | d | de | c | dc | d | d | c | bc | d | c | fde | e | ab | d | d |
| **IS** | a | b | d | e | a | a | c | d | c | bc | a | ab | f | e | c | d | d |
| **SLG** | c | a | d | e | c | c | a | a | a | bc | b | bac | bac | a | c | c | a |

**Supplementary Figure S1.** Schematic representation of specialized plant growth chamber which is equipped with a speaker, light source, and provision for watering from outside.


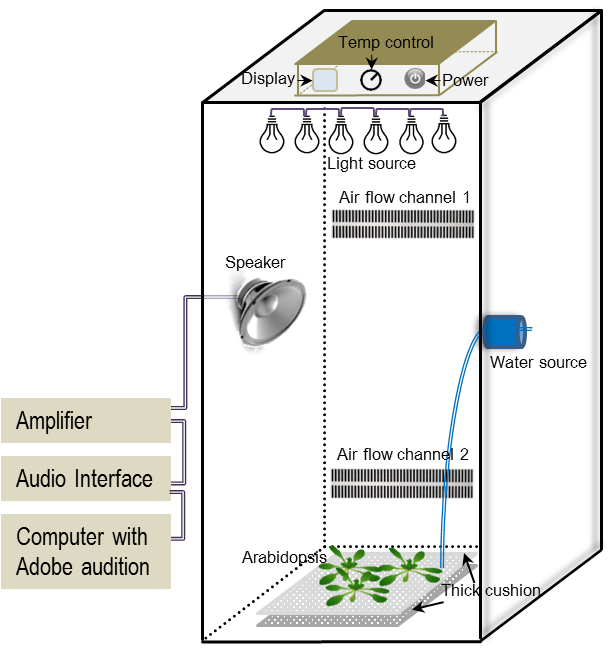


**Supplementary Figure S2.** Expression analysis of SV-regulated genes in various developmental stages. Thirteen tissue samples were analyzed; root (R), stem (S), cauline leaves (CL), young leaves (YL), mature leaves (ML), senescent leaves (SL), young flower (YF), mature flower (MF), young pod (YP), mature pod (MP), ripening pod (RP), imbibed seed (IS) and seedling (slg). Error bar indicates the standard error of means from four biological replications. Statistical analysis (Duncan’s multiple range test, DMRT) are mentioned in Supplementary Table S4.

**Supplementary Figure S3. Quantitative real-time PCR analysis of MS ion channel genes. (A)** Plants were exposed to 500 Hz SV with 100 dB intensity for 5 d in a specialized plant growth chamber. **(B)** Plants were repeatedly touched for 5 d. Expression of each gene in the Arabidopsis treated with mechanical stimuli (gray) was compared with control (black). Expression patterns of these genes were inconsistent between two sets of experiments. Set 1 and 2 indicate the two independent experiments. The y-axis represents the relative expression (%) to *GAPDH.* Error bar indicates the standard error of means from four biological replications. *P*-value ranges are marked by asterisks: * *P*<0.05.


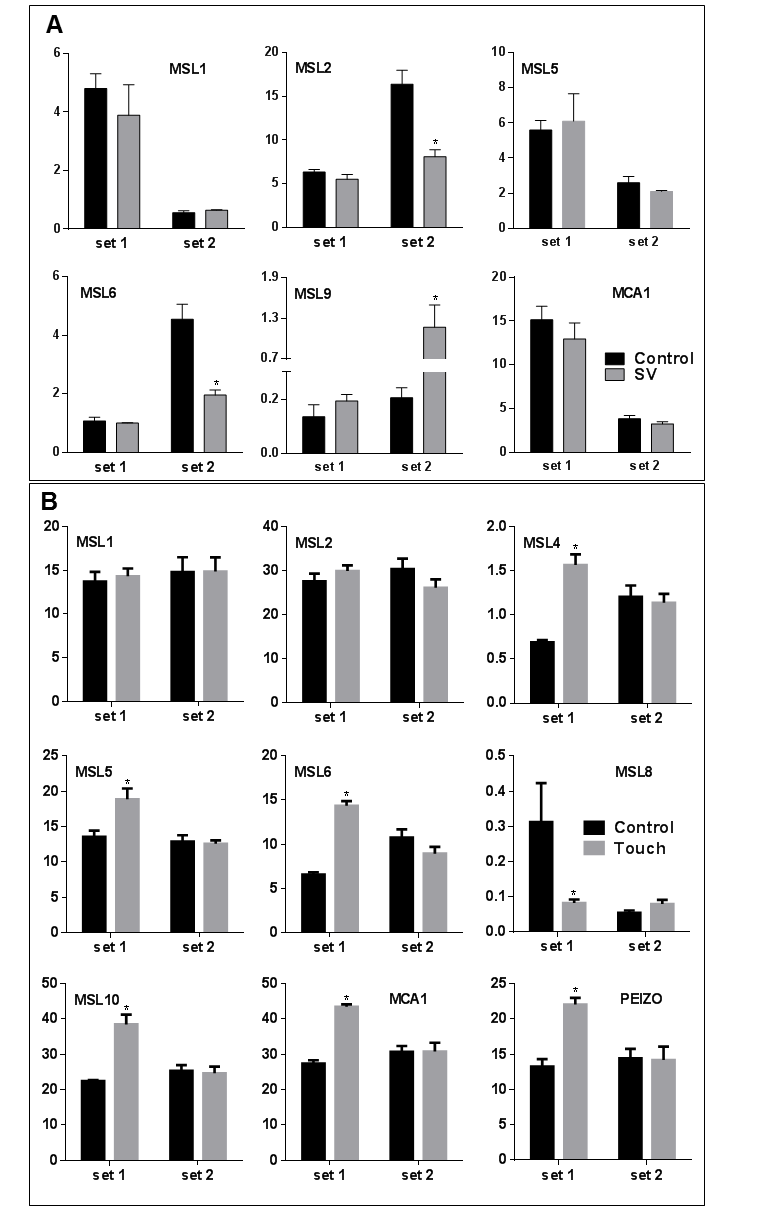

Supplement: Supplementary file 1 [file Data_Sheet_1.docx]
